# Ante-partum Prolapsus of the Cord

**Published:** 1873-11

**Authors:** J. E. O’Brien

**Affiliations:** Scranton, Pa.


ARTICLE IV.—*Ante-partum Prolapsus of the Cord.* By J. E. O'BRIEN, M.D., Scranton, Pa.

Cazeaux speaks always of prolapsus of the cord as an incident of labor only; other authorities whom I have been able to consult, give no intimation of its occurrence at any other time. But the following case shows the accident as occurring at the sixth month of gestation.

January 4th, 1873. Mrs. H., six months pregnant, called me to find an umbilical cord appearing externally and strangulated by the os uteri. Her object in calling me was to know if anything could be done to prevent the miscarriage which previous experience had taught her to expect. She had no living children, but stated that a prolapsus of the cord had occurred to her once before, at the sixth month of gestation, and was followed by abortion. The exciting cause had been a fall, she thought, and, in this second case, exertion in "white-washing."

I could think of no means of restoring the cord, that would offer any hope of saving the child, the means applicable to a case occurring at term seeming to be out of the question, with the os uteri firmly contracted and the fœtus too young to sustain an independent existence. Abortion followed in two days.

My object in publishing this case, is the same as that of the lady in sending for me.

